# Exogenous ethylene application on postharvest oil palm fruit bunches improves crude palm oil quality

**DOI:** 10.1002/fsn3.2423

**Published:** 2021-08-06

**Authors:** Chien Lye Chew, Bee Aik Tan, Jaime Yoke Sum Low, Noor Irma Nazashida Mohd Hakimi, Shwu Fun Kua, Chin Ming Lim

**Affiliations:** ^1^ Sime Darby Plantation Research, R&D Centre – Carey Island Pulau Carey Malaysia; ^2^ Chemical Engineering Discipline School of Engineering Monash University Malaysia Bandar Sunway Malaysia; ^3^ Monash‐Industry Palm Oil Education and Research Platform School of Engineering Monash University Malaysia Bandar Sunway Malaysia; ^4^ Sime Darby Plantation Technology Centre UPM‐MTDC Technology Centre III Serdang Malaysia

**Keywords:** 3‐monochloropropane‐1, 2‐diol esters, chloride, crude palm oil, ethylene, free fatty acid, palm oil mill

## Abstract

Quality and food safety are of paramount importance to the palm oil industry. In this work, we investigated the practicability of ethylene gas exogenous application on post‐harvested oil palm fruit bunches to improve the crude palm oil (CPO) quality. The bunches were first exposed to ethylene gas for 24 hr to induce abscission of palm fruits from bunches. The detached fruits were then subjected to heat treatment, mechanical extraction, clarification and drying to produce CPO. Critical quality parameters of CPO produced, that is free fatty acid, deterioration of the bleachability index and triacylglycerol showed improvement with ethylene gas treatment. Contaminant content that is phosphorus, chloride, iron, and copper also showed a reduction in the CPO derived from ethylene‐treated bunches. These findings corresponded with low levels of contaminants such as 3‐monochloropropane‐1,2‐diol esters and glycidyl esters in refined oil. The implementation strategy and practicability of this method is herein proposed and discussed. Ethylene application not only improves the CPO quality, but could potentially enhance the process sustainability of palm oil mills.

## Graphical abstract

Ethylene (C_2_H_4_) gas application on oil palm fruit bunches improves crude palm oil quality. C_2_H_4_ gas application on palm fruit bunches reduces contaminants in crude palm oil which promote the formation of carcinogenic compounds (3‐chloropropan‐1,2‐diol esters and glycidyl esters) during the refining process. Industrial practicability and implementation strategy are discussed.

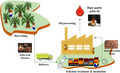

## 1 INTRODUCTION

Palm oil is produced from oil palm fruits, *Elaeis guineensis*, and the extracted oil is known as crude palm oil (CPO) (Sundram et al., 2003). CPO is typically delivered to a refinery for a subsequent processing to produce edible palm oil. CPO contains predominantly neutral oil, also known as triacylglycerol (TAG), along with other minor constituents including free fatty acid (FFA), diacylglycerol (DAG), monoacylglycerol (MAG), moisture, solid impurities, phospholipids, antioxidants (such as carotenes, tocopherols, sterols), lipid oxidation products, metal contents, and chlorides (Chew et al., 2021; Maclellan, 1983; Mancini et al., 2015). These minor constituents are impacting the overall quality of the CPO produced. In the palm oil industry, CPO quality specifications set by the Palm Oil Refiners Association of Malaysia (PORAM) are commonly used as a standard quality requirement for the trading of CPO between millers and refiners, illustrated in Table 1 (PORAM, 2000). According to the standard, the FFA indicates the hydrolytic stability of the oil and the deterioration of bleachability index (DOBI) implies the acceptance level in the refining process. Lastly, the moisture and impurities (M&I) determine the purity of the CPO produced (Ariffin, 2001; Chong, 2012).

**TABLE 1:** Quality requirement for CPO in PORAM specification (PORAM, 2000)

Characteristics	PORAM standard
Free fatty acid (as palmitic), % max	5.0
Moisture and impurities, % max	0.25
Degree of Bleachability Index, minutes	2.3

Abbreviation: PORAM, Palm Oil Refiners Association of Malaysia.

Generally, the CPO is susceptible to lipid oxidation, causing oil instability and reduces shelf life (Frega et al., 1999). During production, CPO is exposed to oxygen and oxidising agents that degrade the oil through instantaneous free radical chain reaction known as autoxidation (Berger & Hamilton, 1995). High FFA levels in the CPO could promote lipid oxidation, which lead to oxidised compound formation. The oxidised compounds, in turn leads to poor oil flavour and affect palatability. (Choe & Min, 2006; Chong, 2012; Wai et al., 2009). Therefore, FFA is a critical parameter to measure the quality of the CPO.

FFA formation in the oil is predominantly caused by sub‐optimal harvesting and handling techniques of fresh palm fruit bunches (FFB). During the FFB transportation step, the palm fruits may incur injury, resulting in the oil body membrane rupturing and exposing the oil to the lipase enzymes adjacent to the outer surface of lipid droplets (Morcillo et al., 2013). The lipase catalyses lipid through a hydrolysis reaction and breaks TAG into partial acylglycerol and FFA (Wong et al., 2016).

The presence of impurities could also degrade the quality of the CPO. As an example, pro‐oxidant metals (i.e. iron and copper) in the CPO promote thermal autooxidation (Chong, 2012; Das & Pereira, 1990; Willems & Padley, 1985). Likewise, studies also confirmed that the chloride content in CPO was a major precursor for process contaminant known as 3‐monochloropropane‐1,2‐diol esters (3‐MCPDE). This contaminant is formed during refining process as the result of a chemical reaction in the deodorisation stage (Craft et al., 2012; Šmidrkal et al., 2016; Tiong et al., 2018). Meanwhile, glycidyl ester (GE), a heat‐induced contaminant, is formed by presence of partial acylglycerols under high temperature during oil refining process (Craft et al., 2012). During application, oil along with these contaminants could be absorbed by the food (Chew, Ab Karim, Quek, et al., 2021).

Ethylene is one of the most widely used phytohormone in the agriculture sector. Its application promotes ripening in climacteric fruits, regulating the colour changes of fruits, reducing chlorophyll and increasing carotenoids, in addition to being important in abscission and senescence in plants (Iqbal et al., 2017). Oil palm fruits respond to endogenous and exogenous ethylene as they exhibit climacteric fruits characteristics (Tranbarger et al., 2011). Aside from ripening, enhancement of abscission in agriculture is also of interest. One study has shown that this phytohormone could potentially be used to induce palm fruits ripening and abscission from bunches to increase total oil content (Nualwijit & Lerslerwong, 2014). However, there is limited research on the effect of ethylene application on the quality of the palm oil produced.

In this research, we explore the feasibility of using the exogenous ethylene (C_2_H_4_) gas to accelerate palm fruit detachment from bunches with a particular goal to reduce the FFA and chloride contents in CPO. It was hypothesised that after ethylene application on FFB the detached fruits separated from the bunches will incur less damage (during conveyance) and consequently have lower FFA formation during milling. Additionally, the detached fruits will be processed without the chloride‐rich empty fruit bunches (EFB), and this could potentially reduce the amount of the chloride content in the CPO and, subsequently, 3‐MCPDE in refined oil. The outcome of this research could enable the development of new processes and improved quality of CPO in the mill.

## 2 MATERIALS AND METHODS

### 2.1 Materials

All reagents and solvents used in this study were analytical grade unless specified. Iso‐octane (2,2,4‐trimethylpentane) used for DOBI and carotene analyses were of mass spectrometry grade. FFB were collected from an estate in Selangor (Sime Darby Plantation, Malaysia).

### 2.2 Exogenous ethylene application procedure

Approximately 8 tonnes of ripe FFB with an average weight of 15.0–20.0 kg were collected from the estate. Standard ripe FFBs were determined based on the number of detached fruits (>10) on the ground before harvesting (Chew, Low, et al., 2021; Chew, Ng, et al., 2021). The FFB were then placed in a closed modified 10 tonnes bin with a dimension of 2175 mm (width) × 5,490 mm (length) × 1,550 mm (height) and delivered to a nearby Sime Darby Plantation oil mill. Approximately 125 ppm per 1,000 kg FFB of the exogenous ethylene (Gaslink, Malaysia) was injected into the container and incubated for 24 hr. Separately, a set of FFB were prepared without the introduction of exogenous ethylene as control samples. The experiments were repeated for five times with different set of FFB under the same process condition (i.e. FFB weight, FFB ripeness, ethylene concentration, incubation time and equipment).

### 2.3 Production crude palm oil

**FIGURE 1:**
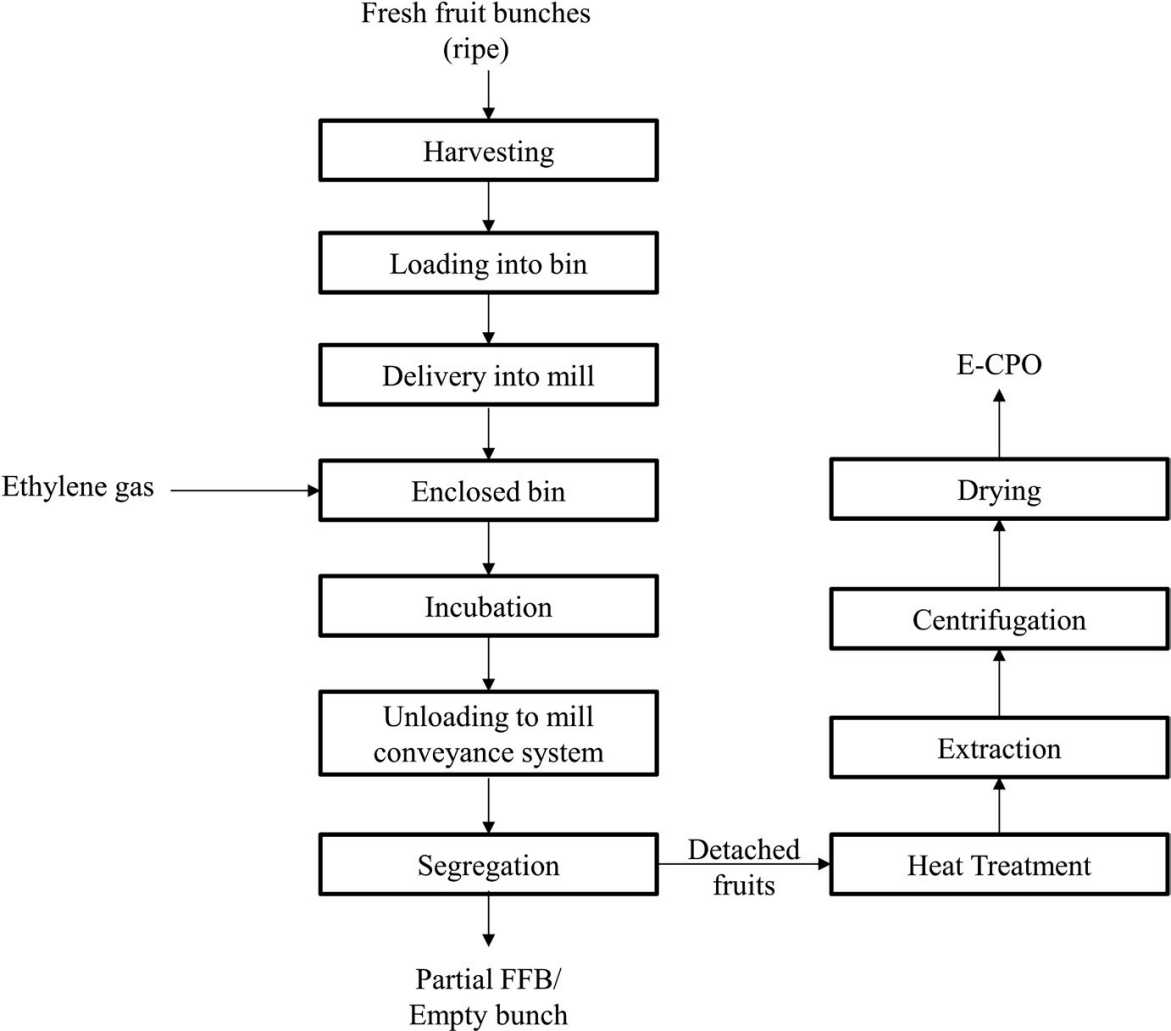
Block diagram of the E‐CPO production

After 24 hr of ethylene treatment on FFB, the detached fruits were separated from bunches with a mechanical segregator present in the mill conveyor system and then subjected to heat treatment in a mill steriliser at 140°C for 85 min. After heat treatment, the crude oil mixture was manually extracted from the palm fruits with a mechanical press. The mixture was then centrifuged and dried to obtain the ethylene‐treated crude palm oil (E‐CPO) with moisture content below 0.2% and kept at a cold temperature (4°C) for further analysis. The detailed procedure of the E‐CPO production is illustrated in Figure 1. A similar approach was used to produce the control crude palm oil (C‐CPO) from the control FFB.

### 2.4 Production of refined palm oil

Physically refined C‐CPO and E‐CPO were denoted as C‐RPO and E‐RPO, respectively. The refining process involved three major stages, namely, degumming, bleaching, and deodorisation. For degumming, the oil was dosed with 0.06% phosphoric acid (85%, v/v), and the mixture was mixed under vacuum for 20 min at the controlled temperature of 85°C. Approximately, 1% (w/w) bleaching earth was added to the degummed oil and mixed for 30 min under at 95°C. Then, the bleached oil was filtered using filter paper before deodorisation steps. During the deodorisation, the bleached oil was heated up to 260°C, and sparging steam was introduced for 90 min.

### 2.5 Oil quality analysis

The FFA content in the CPO was determined according to the AOCS official method, Ca 5a‐40 (AOCS, 2017). Meanwhile, the carotene content and DOBI value were analysed according to the MPOB Test Method p2.9:2004 and p2.6:2004, respectively (MPOB, 2004). The oil oxidation is determined through peroxide value (PV) and anisidine value (AnV) using the AOCS official method, Cd 8–53 and Cd 18–90, respectively (AOCS, 2017). The total oxidation (TOTOX) was calculated with the summation of 2PV and AnV. Meanwhile, the iodine value (IV) indicates the degree of unsaturation of a fat or oil. Determination of IV was carried out according to the MPOB Test Method p3.2:2004 (MPOB, 2004). Calculation of the IV was carried out and expressed as g of iodine/100 g of oil.

The impurities (insoluble solid) of CPO were determined by the ISO 663:2007(E) method (International Standard, 2007). Meanwhile, the moisture content of the CPO sample was determined using a Karl Fisher Titrator according to the AOCS official methods, Ca 2e‐84 (AOCS, 2017). This method determines the actual water content of fats and oils by titration with Karl Fischer reagent, which reacts quantitatively with water. The Karl Fisher reagent (HYDRANAL™ ‐ Coulomat A and CG) was used to titrate with water content in oils and fats to determine the water content of the sample. Both solid impurities and moisture were combined and denoted as M&I.

### 2.6 Acylglycerols composition analysis

The oil sample (0.05 g) was dissolved in n‐hexane (5 ml) and then analysed for acylglycerol composition using gas chromatography (Model: Clarus 500; Perkin, Elmer, Waltham, Massachusetts, USA) equipped with a flame ionisation detector (Chew, Ab Karim, Quek, et al., 2021). The acylglycerols were separated using a SP2380 (Supelco; Bellefonte, Pa., USA) capillary column (0.25 cm i.d. × 30 cm × 0.2 μm). The temperature programme was as follow: 120–290°C for 25°C/min, then heated from 290–340°C for 10°C/min and from 340–360°C for 15°C/min (hold for 2 min). The injector and detector temperature were set at 100°C and 370°C. The carrier gas was nitrogen at 45 ml/min with the injection volume at 1 μl.

### 2.7 Contaminant analysis

The level of trace contaminants, that is phosphorus, iron, and copper, in the CPO samples were determined. The phosphorus content was determined using the AOCS Official Method Ca 12b‐92 (AOCS, 2017). The CPO samples were diluted with blank oil in a mass ratio of 1:5. The phosphorus content was determined using an atomic absorption spectrometer (AAS) at a wavelength of 213.6 nm. On the other hand, copper and iron were determined by AOCS Official Method Ca 15–75 (AOCS, 2017). CPO samples dissolved in methyl isobutyl ketone (MIBK) were analysed for metals by direct aspiration. The copper and iron in CPO samples were calculated from a calibration curve, whereby the calibration curve was developed based on MIBK solutions of organometallic reference compounds solubilised in purified vegetable oil.

The total chloride content was analysed with a total chloride analyser (Mitsubishi Chemicals NSX > 2,100 series), according to the ASTM D 4929‐04 Method B (ASTM, 2010). Finally, the presence of 3‐MCPDE and GE in oils were detected quantitatively by gas chromatography–mass spectrometry (GC–MS) based on the AOCS Official Method Cd 29a‐13 (AOCS, 2017). The contaminants were determined by alkaline‐catalysed alcoholysis reaction using sodium methoxide in methanol and derivatisation with phenylboronic acid (PBA).

### 2.8 Statistical analysis

All the oil quality analyses were conducted in triplicate, and the average results represented each set of experiment. The final results (of 5 set experiment) were expressed as mean values together with their standard deviation. The statistical analysis of variance (ANOVA) was carried out using GraphPad Prism (GraphPad Software Inc.) program, and the differences were considered statistically significant when *p* < .05.

## 3 RESULTS AND DISCUSSION

### 3.1 Critical quality parameters of ethylene‐treated FFB produced palm oil

FFA content, DOBI, moisture, and impurities content are essential indicators of CPO quality. In this study, the FFA content of E‐CPO was 63% lower than that of C‐CPO (Table 2). Similarly, there was a significant (*p* < .05) improvement (26%) in DOBI value observed in E‐CPO compared to C‐CPO, which favours the refining process. The moisture and impurities (insoluble dirt) of E‐CPO and C‐CPO were well below 0.25% (w/w), indicating that the produced oil is within the specification limit.

**TABLE 2:** Critical quality parameters of C‐CPO and E‐CPO. Results represent the means ± standard deviation of the mean value (*n* = 5)

Characteristic	C‐CPO	E‐CPO
FFA (%)	3.77 ± 0.40^a^	1.38 ± 0.49^b^
DOBI	2.95 ± 0.19^a^	3.73 ± 0.10^b^
M&I (%)	0.15 ± 0.02^a^	0.16 ± 0.03^a^
PV (meq O_2_/kg)	1.09 ± 0.88^a^	0.64 ± 0.44^b^
AnV	2.90 ± 0.46^a^	2.36 ± 0.37^b^
TOTOX	5.46 ± 2.18^a^	3.64 ± 1.42^b^
Carotene (ppm)	591 ± 26^a^	635 ± 47^b^
IV (I_2_/100g)	52.92 ± 0.72^a^	53.04 ± 0.71^a^
TAG (%)	94.61 ± 1.27^a^	97.79 ± 1.15^b^
DAG (%)	2.06 ± 0.34^a^	1.38 ± 0.20^b^
MAG (%)	0.11 ± 0.05^a^	0.00^b^
Iron, Fe (ppm)	6.26 ± 0.95^a^	1.48 ± 1.47^b^
Copper, Cu (ppm)	0.14 ± 0.05^a^	0.03 ± 0.02^b^
Phosphorus, P (ppm)	19.48 ± 4.79^a^	8.12 ± 1.95^b^
Chloride, Cl (ppm)	3.03 ± 0.58^a^	1.46 ± 0.39^b^

Note

Mean values with different letters in the same row are statistically different (*p* < .05).

Abbreviations: 3‐MCPDE, 3‐monochloropropane‐1,2‐diol esters; AnV, anisidine value; C‐CPO, crude palm oil produced from ethylene‐treated palm fruits bunches; DAG, diacylglycerol; DOBI, deterioration of bleachability index; E‐CPO, crude palm oil produced from ethylene‐treated palm fruits bunches; FFA, free fatty acid; GE, glycidyl esters; IV, iodine value; M&I, moisture and solid impurities; MAG, monoacylglycerol; PV, peroxide value; TAG, triacylglycerol; TOTOX, total oxidation.

The influence of ethylene treatment of fruits on the CPO oxidative stability was examined by determining the level of primary and secondary oxidation products. The PV and AnV level of E‐CPO was significantly (*p* < .05) lower than that of C‐CPO. For a better representation of oxidative oil stability, TOTOX is used. Although TOTOX is not specified in any standards, it is widely used for quality monitoring (Esfarjani et al., 2019; Xu et al., 2015). The results showed that TOTOX in E‐CPO was 3.64 ± 1.42 compared to 5.46 ± 2.18 in C‐CPO. Meanwhile, the results showed that the carotene (antioxidant) content in E‐CPO (635 ± 47 ppm) was comparable to C‐CPO (591 ± 26 ppm) which fall into the typical carotene range in palm oil between 500 and 700 ppm (Chew, Ab Karim, Kong, et al., 2021; Chew, Ab Karim, Quek, et al., 2021; Chong, 2012).

The influence of ethylene‐treated FFB on the acylglycerols composition of oil was also investigated. The TAG in the E‐CPO was significantly (*p* < .05) higher than that of C‐CPO. The DAG in E‐CPO was lower than that of C‐CPO, with no significant differences (*p* > .05). Meanwhile, no MAG was detected in E‐CPO, while a small amount of MAG was detected in C‐CPO. The typical range for IV in palm oil is between 52 and 55 (Chong, 2012). The results showed that the IV of E‐CPO was comparable to C‐CPO and within the recommended range. This finding showed the ethylene treatment did not alter the palm oil's acylglycerols composition or the degree of unsaturation.

Trace contaminants present in the E‐CPO were investigated. In this study, the contents of trace metals such as iron and copper in E‐CPO were significantly (*p* < .05) lower than those in C‐CPO. Similarly, contaminants such as phosphorus and chloride content in E‐CPO were substantially lower (*p* < .05) than those of C‐CPO. The obtained results demonstrated lower residues of contaminants in the CPO produced from ethylene treatment. Subsequently, the E‐CPO was refined and tested for 3‐MCPDE content and GE (Table 3). The results showed that the 3‐MCPDE and GE in E‐RPO were 77% and 57% lower than the C‐RPO, respectively.

**TABLE 3:** 3‐MCPDE and GE content in C‐RPO and E‐RPO. Results represent the means ± standard deviation of the mean value (*n* = 5)

Characteristic	C‐RPO	E‐RPO
3‐MCPDE (ppm)	2.48 ± 0.33^a^	0.58 ± 0.17^b^
GE (ppm)	1.36 ± 0.28^a^	0.58 ± 0.07^b^

Note

Mean values with different superscript letters in the same row are statistically different (*p* < .05).

Abbreviations: 3‐MCPDE, 3‐monochloropropane‐1,2‐diol esters; C‐RPO, refined palm oil produced from control palm fruits bunches; E‐RPO, refined palm oil produced from ethylene palm fruits bunches; GE, glycidyl esters.

It is worth highlighting that a commercial scale batch of palm fruit bunches (8 MT) was used for this study rather than single or small sample sizes (FFB) in order to ensure that a practical experiment could be devised and results were practical to mill processes. Prior to this study, a small‐scale study was conducted with a single palm fruit bunch under controlled conditions to produce E‐CPO and C‐CPO. However, the results obtained from a small‐scale study were inconclusive due to multiple points of diversion from typical commercial processing (data are not shown). For instance, the impact of the mill conveyance system on FFB and detached fruits to the quality of the CPO produced were not replicated in the small‐scale study. The effect of the mill facilities and processing units on the oil quality were discussed in the next section.

### 3.2 Postulation of mechanisms

**FIGURE 2:**
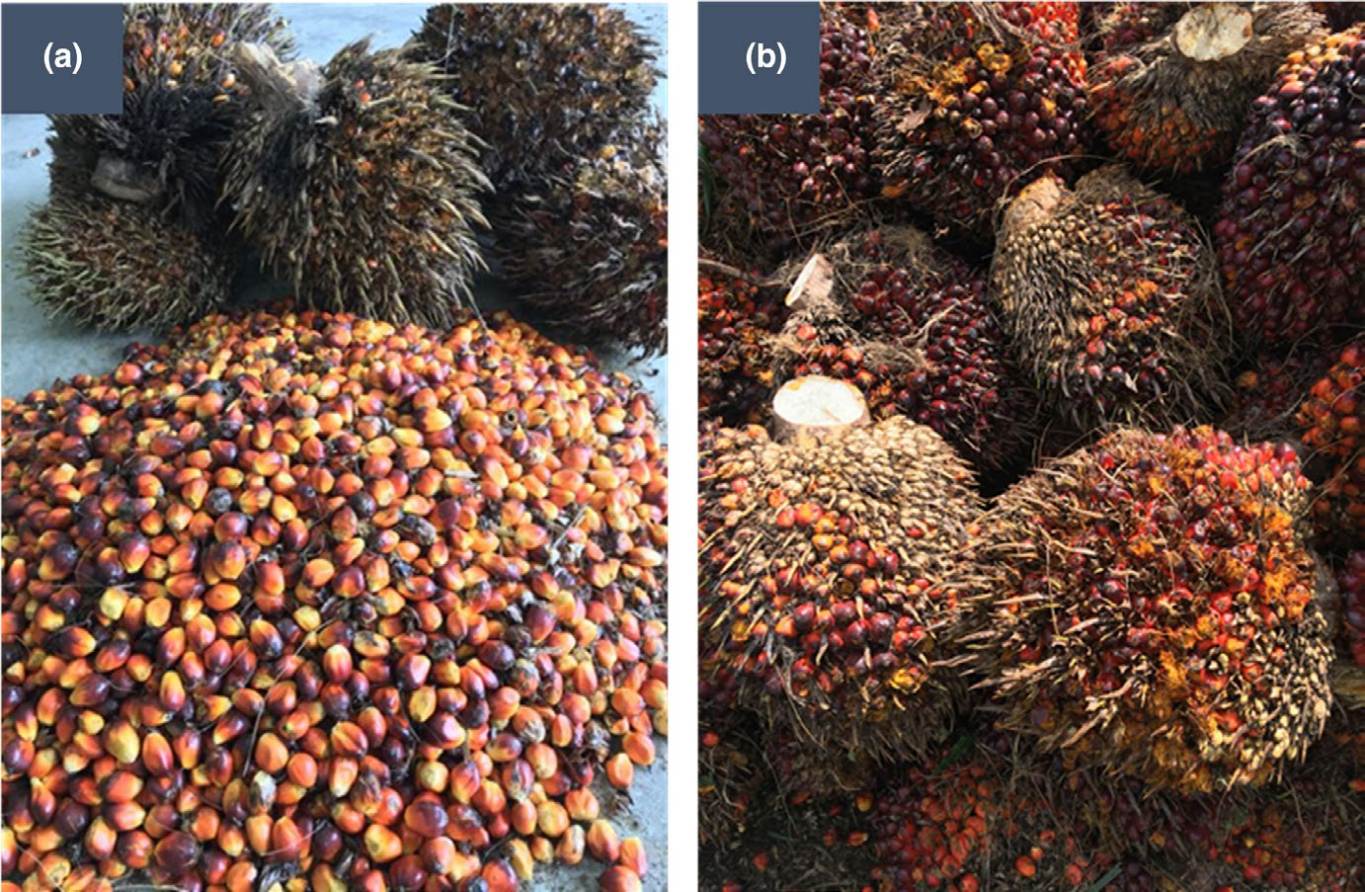
Visual observation of (a) outer layer palm fruits detached with minimal fruit damage after ethylene treatment and (b) damaged fruits on FFB due to postharvest handling and transport

Critical parameters such as FFA, DOBI, and AnV were highlighted as main indicators to oxidative stability. It was presented that E‐CPO had a better overall quality than standard CPO (C‐CPO) parameters. FFA formation was largely attributed by lipolytic lipase enzymes which were released during cell wall ruptures and caused hydrolysis of TAG sequentially to DAG, MAG, and FFA (Wong et al., 2016). As a result, the high amount of TAG with a low amount of partial acylglycerols in the E‐CPO compared to C‐CPO due to lower FFA formed. In this work, the FFA content in the E‐CPO was found to be lower than that in C‐CPO despite there may be a dilution effect of the inner fruits of the FFB which contains very low FFA. Low FFA in E‐CPO was attributed by minimal palm fruits damage during handling. This finding was supported by visual observation of detached palm fruits (Figure 2), where less visible damage was observed to the palm fruits after detached from ethylene gas–treated FFB. A study showed that the severity of the palm fruits damage was directly correlated (*r* = .91) to the FFA of its extracted oil (Hadi et al., 2009). The highest point of palm fruits damage was located at the mill loading ramp, where the FFB was unloaded to the mill conveyance system (Krisdiarto & Sutiarso, 2016). Ethylene treatment will induce palm fruits abscission from spikelets, resulting in a weight reduction of the bunches (Nualwijit & Lerslerwong, 2014). If the detached fruits are to be sterilise together with the partial stripped FFB, the lower FFB weight resulting from the ethylene treatment potentially reduces the impact force that damage the fruits during unloading steps and subsequent crop conveyance, resulting in lower fruit damage. This postulation is supported by the evidence of the fruit detachment (with ethylene treatment) was between 20% and 25% of its bunch weight, resulting in the reduction of FFB weight (data are not shown). Meanwhile, there is no reduction of bunch weight for control samples.

Pro‐oxidant metals (i.e. iron and copper) present in CPO reduces oxidative stability (Choe & Min, 2006; De Leonardis et al., 2016). It was hypothesised that the reduction of FFB weight reduces its impact on the mill conveyance system, resulting in lower wear and tear of the equipment, and most likely low amount of these metals was picked up during processing. Through ethylene application, the palm fruits detached from FFB reducing the overall bunch weight to produce E‐CPO with low iron and copper contents. Nevertheless, a more in‐depth study is required to confirm this finding. A previous study has shown that the removal of EFB reduces most of the contaminants such as chloride and phosphorus are in the produced CPO (Chew & Norliza, 2021). In the conventional palm oil mill processing, the removal of the EFB could only be done after sterilisation where sterilisation induces fruit detachment from its bunch. As the result, the chloride and phosphorus from the EFB could be carried over during sterilisation. The application of ethylene enables the detachment of palm fruits and segregation of its bunch at an earlier stage, before sterilisation. Therefore, the source of contaminants, the bunch was removed during processing to produce E‐CPO with low chloride and phosphorus contents.

A low PV in E‐CPO was possibly due to lower content of metals involved to form the peroxides than C‐CPO. Iron and copper which are lower in E‐CPO reduce the activation energy of the autoxidation (initiation stage) and increase the lipid oxidation rate (Jadhav et al.,^1996^). Subsequently, lipid peroxides were decomposed to form the free radicals by a redox cycling pathway. These free lipid radicals lead to the formation of new peroxides in the presence of oxygen which increase the PV value. The AnV was also lower in E‐CPO indicating no significant breakdown of pre‐existing peroxides in the oil. Lower iron and copper in E‐CPO would lead to reduced formation of secondary products such as the aldehydes, ketones, alcohols, and hydrocarbons from the breakdown of pre‐existing peroxides and lead to increased value of AnV (Choe & Min, 2006; Mozuraityte et al., 2016). Non‐metal impurity such as phosphorus could also affect the CPO's oxidative stability. Low content of phosphorus in E‐CPO indicates low content of phospholipids (Carelli et al., 2002). The phospholipids present in the oil could accelerate lipid oxidation due to its hydrophilic group that decreases the oil surface tension and increases oxygen diffusion (Choe & Min, 2006). Similarly, FFA, DAG, and MAG which contain hydroxyl groups could also increase the lipid oxidation rate (Choe & Min, 2006). It was also noted that shorter heat treatment duration to the detached palm fruits likely reduced the oxidative stability of oil produced. Studies showed that oil processing with prolonged heat treatment at high temperature caused oil oxidation (Khor et al., 2019; Oboh et al., 2014; Velasco & Dobarganes, 2002). This finding is strengthened by the increase of DOBI in E‐CPO, which means oil deterioration due to oxidation was slowed down with low FFA and contaminants level. In short, the ethylene treatment reduces the prooxidants and improves the overall E‐CPO quality.

3‐MCPDE and GE are the potential carcinogenic contaminants present in most vegetable oils, including palm oil (EFSA, 2016; IARC, 1997). From the study, we observed a reduction of the chloride content in E‐CPO. This finding could be attributed by the separation of the palm fruits from EFB during processing. A study suggested that fibrous materials such as EFB enriched the chloride content in the CPO produced (Matthäus & Pudel, 2013). Meanwhile, another study confirmed the role of EFB in enhancing the chloride content in CPO (Chew & Norliza, 2021). Similarly, the reduction of phosphorus was due to the removal of phosphorus‐rich EFB during oil processing. The oil samples recovered from EFB contain between 80 and 124 ppm of phosphorus (data are not shown). Apart from chloride as the most significant contributor, acidity, DAG, and MAG were associated with the formation of 3‐MCPDE (Craft et al., 2012; Sim et al., 2018; Šmidrkal et al., 2016). During refining process at high temperature, chloride can react with the glycerol backbone of lipids to produce 3‐MCPDE (Craft et al., 2012; Šmidrkal et al., 2016; Tiong et al., 2018). The lower FFA level in E‐CPO was translated to a lower acidity of the oil which help to reduce the formation of 3‐MCPDE (Ramli et al., 2015). A lower content of partial aycylglygerols specifically DAG in E‐CPO often resulted in lower amount of 3‐MCPDE and GE in its refined oil (E‐RPO) (Craft et al., 2012; Šmidrkal et al., 2016). The results above were an essential indication that ethylene treatment on palm fruits had improve the palm oil quality.

### 3.3 Industrial Practicability

**FIGURE 3:**
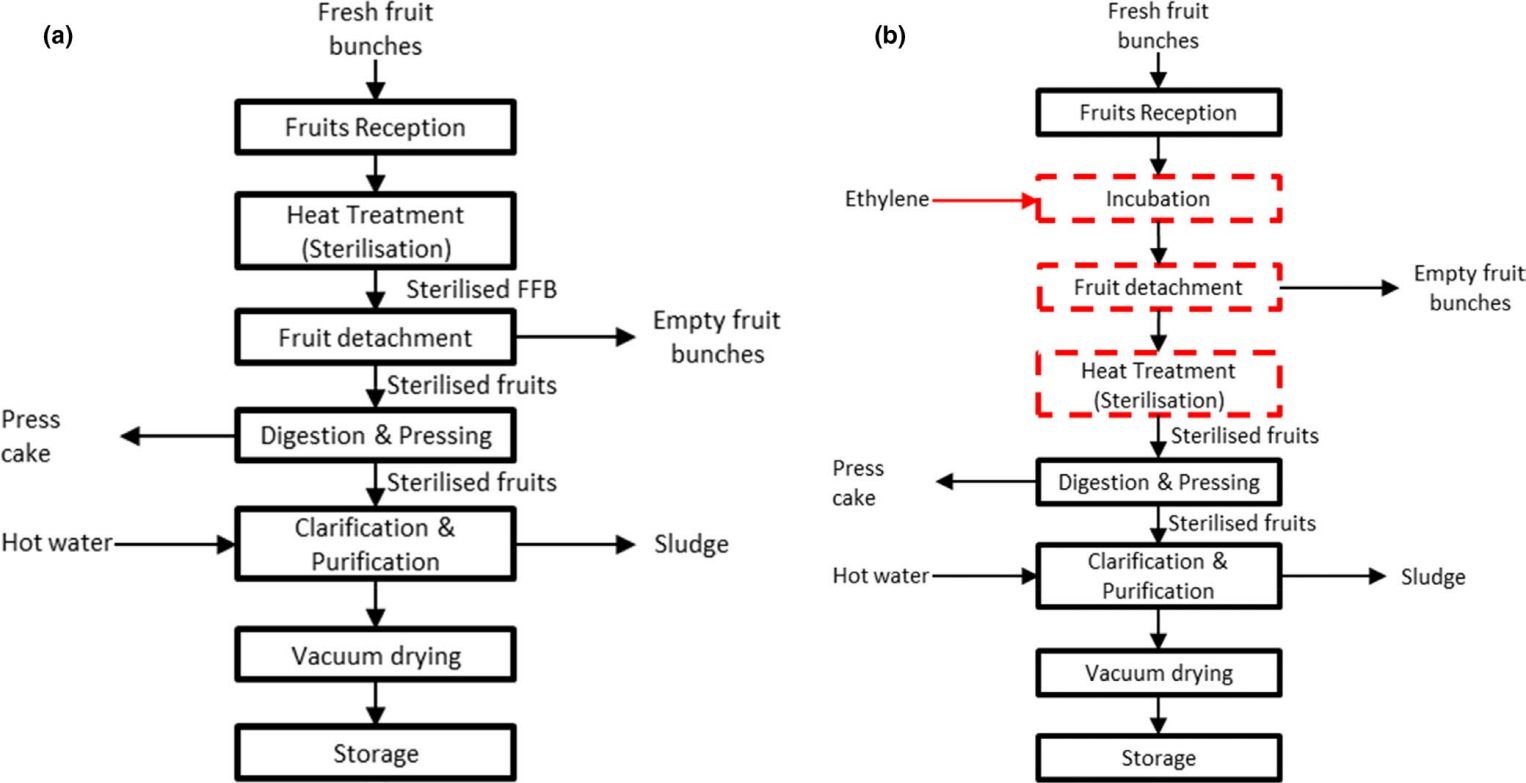
Process flow of (a) conventional palm oil mill; (b) proposed implementation strategy for ethylene application

Besides quality improvement, the ethylene treatment offers a potential reduction of energy used for heat treatment (sterilisation). In the conventional process (Figure 3a), intensive amount of energy (in the form of steam) is required to heat the FFB. Approximately 200 kg of steam is necessary to heat‐treat 1 tonne of FFB, which eventually contributed to the mass amount of wastewater (Chew, Low, et al., 2021; Chew, Ng, et al., 2021; Yap et al., 2020). The heat treatment's primary function is to deactivate the lipase enzyme (>50°C) for arresting the FFA rise and facilitate the fruit detachment from its bunches. Therefore, the conventional heat treatment temperature was at 140°C to ensure sufficient heat penetration of FFB that has a wide range of weight and bunch architecture (Chew, Low, et al., 2021). The heat treatment could be reduced in terms of time and temperature by introducing the ethylene treatment as the palm fruits would already be detached from the spikelets of the FFB before sterilisation. Therefore, the amount of weight input into the steriliser would greatly be reduced. Consequently, the production of wastewater and fuel consumption of the mill could be reduced significantly.

Conventional mill process requires a big mechanical rotating drum to facilitate the fruits detachment after the heat treatment stage (Chew, Ng, et al., 2021; Prasertsan & Prasertsan, 1996). The authors proposed a simple segregating system along with the mill conveyance system before the heat treatment after ethylene gas–aided detachment step. This method could ensure only detached palm fruits undergo heat treatment without empty fruit bunches and proceed to pressing station. Separation of bunches could lead to a better mill process with lower wear and tear, resulting in lower sources of contamination. This technology could also re‐engineer the milling process flow and address its long‐standing issue by converting the semi‐continuous pressurised batch heating process to a continuous milling process (Figure 3b). The pressurised system of the semi‐continuous batch heating process is known to be hazardous and consumed high energy at an extended period of time (Omar et al., 2018; Sivasothy et al., 2005; Vincent et al., 2014). It is worth noting that there is a development of a continuous sterilisation process at atmospheric pressure at the mill. This continuous system is incorporated with bunch splitter that cuts FFB into half before heat treatment, resulting in high FFA content in the produced CPO (Omar et al., 2018; Sivasothy et al., 2005). Furthermore, this method consumed greater steam at 300 kg/tonne FFB than conventional pressurised heat treatment (Chew, Low, et al., 2021; Omar et al., 2018). Ethylene application which produces detached palm fruits could potentially dismiss the bunch splitter's use and lowering FFA in CPO produced. Steam consumption of the continuous system could be reduced because the incoming material mass (detached fruits) is smaller than FFB. This method could improve the sustainability of palm oil processing.

## 4 CONCLUSION

In this work, we have shown that ethylene application is feasible in improving crude palm oil quality. All the critical quality parameters of the crude palm oil, that is free fatty acid, oxidative stability and deterioration of bleachability index, showed improvement after ethylene treatment on fresh fruit bunches to induce abscission of the fruits. The ethylene treatment on fresh fruit bunches was able to reduce the undesirable non‐metal elements (chloride and phosphorus) by removing EFB from the process. This technique could improve the quality of crude palm oil and food safety by reducing the level of compounds (free fatty acid, diacylglycerol and chloride contents) associated with the formation of 3‐monochloropropane‐1,2‐diol esters and glycidyl esters during refining. Interestingly, the trace metal contaminants specifically iron and copper were also reduced due to empty fruit bunches removal. In current mill process, incorporation of washing fresh fruit bunches in line to remove the contaminants is still not feasible due to large capital expenditure and limited technology. If mills could process only detached fruits from ethylene treatment FFB, there would be around 40% of mass reduction contributed by empty fruit bunches. This would result in smaller ramp space, a more compact conveyor system, and removal of threshing process after sterilisation. Implementation of this technology in palm oil mill could also improve the practicability of the continuous sterilisation with lower energy consumption and minimise wastewater generated. This ethylene application is a practical and innovative solution for the palm oil industry that could revamp the process flow and perhaps towards a smaller footprint mill facility.

## CONFLICTS OF INTEREST

The authors declare no conflict of interest.

## AUTHOR CONTRIBUTIONS

**Chien Lye Chew:** Conceptualization (equal); Formal analysis (equal); Investigation (equal); Methodology (equal); Visualization (equal); Writing‐original draft (lead); Writing‐review & editing (equal). **Bee Aik Tan:** Investigation (equal); Visualization (equal); Writing‐original draft (equal); Writing‐review & editing (equal). **Jaime Yoke Sum Low:** Data curation (equal); Methodology (equal); Writing‐original draft (equal); Writing‐review & editing (supporting). **Noor Irma Nazashida Mohd Hakimi:** Data curation (equal); Methodology (equal); Supervision (lead); Visualization (equal). **Shwu Fun Kua:** Conceptualization (supporting); Investigation (equal); Methodology (equal); Validation (equal); Visualization (equal). **Chin Ming Lim:** Project administration (lead); Resources (lead); Supervision (lead); Writing‐review & editing (supporting).

## Data Availability

The data that support the findings of this study are available from the corresponding author upon reasonable request.
